# The human asparaginase enzyme (ASPG) inhibits growth in leukemic cells

**DOI:** 10.1371/journal.pone.0178174

**Published:** 2017-05-24

**Authors:** Stefania Belviso, Rodolfo Iuliano, Rosario Amato, Nicola Perrotti, Miranda Menniti

**Affiliations:** 1 Department of Experimental and Clinical Medicine, University Magna Graecia of Catanzaro, Catanzaro, Italy; 2 Department of Human Health, University Magna Graecia of Catanzaro, Catanzaro, Italy; University of South Alabama Mitchell Cancer Institute, UNITED STATES

## Abstract

The human protein ASPG is an enzyme with a putative antitumor activity. We generated in bacteria and then purified a recombinant GST-ASPG protein that we used to characterize the biochemical and cytotoxic properties of the human ASPG. We demonstrated that ASPG possesses asparaginase and PAF acetylhydrolase activities that depend on a critical threonine residue at position 19. Consistently, ASPG but not its T19A mutant showed cytotoxic activity in K562, NALM-6 and MOLT-4 leukemic cell lines but not in normal cells. Regarding the mechanism of action of ASPG, it was able to induce a significant apoptotic death in K562 cells. Taken together our data suggest that ASPG, combining different enzymatic activities, should be considered a promising anti-cancer agent for inhibiting the growth of leukemia cells.

## Introduction

ASPG (Uniprot code Q86U10), also named 60 kDa Lysophospholipase, is a protein that should have asparaginase, lysophospholipase, transacylase and PAF (platelet-activating factor) acetylhydrolase activities.

The catalytic domain of ASPG is located in its N-terminal part that contains also an ankyrin repeat, whereas the C-terminal region of the protein includes four ankyrin repeats. The rat form of ASPG was characterized and asparaginase, lysophospholipase, and PAF acetylhydrolase activities were demonstrated [1]. The asparaginase activity of the N-terminal region of human ASPG was described in detail [2].

We previously reported that ASPG is a new molecular partner of the serine-threonine kinase SGK1 [3]. The main effect of ASPG that we observed in eukaryotic cells was the down-regulation of the epithelial sodium channel (ENaC) activity, which is a feature associated with the decrease of cell malignancy [4]. We also suggested that ASPG, through its lysophospholipase activity, can play a critical role in the control of cell proliferation mediating the conversion of lysophosphatidylinositol into glycerophosphoinositol, which is an important intracellular messenger derived from RAS pathway [3]. These findings and the enzymatic activities of ASPG suggest that it represents a key element in the inhibition of tumor cell growth.

The therapeutic role of L-asparaginases is based on their ability to hydrolyze L-asparagine into L-aspartate and ammonia, depriving tumor cells of a critical metabolite. More specifically, leukemia cells require large amounts of L-asparagine in order to sustain the rapid malignant growth. In contrast, the supply of L-asparagine is dispensable for healthy cells that can synthesize the amino acid in sufficient amounts by their L-asparagine synthetase (ASNS). Clinical trials demonstrated the effectiveness of L-asparaginases in the treatment of pediatric and adult acute lymphoblastic leukemia (ALL) patients [5] and the use of L-asparaginases appears to be promising in the therapy of other hematologic malignancies [6] and solid tumors [7]. All commercial L-asparaginases are bacterial-derived enzymes that cause immunological reactions neutralizing the therapeutic effects and inducing adverse reactions in more than 50% of cancer cases [8]. Thus, the adoption of a human asparaginase enzyme could overcome the side effects associated with the administration of bacterial proteins.

PAF-AH (PAF acetylhydrolase) catalyzes the biochemical conversion of PAF into the biologically inactive lyso-PAF by removing the acetyl group at the sn-2 position. It also metabolizes glycerophospholipids containing short, oxidized, and/or fragmented sn-2 acyl group that are typically generated during inflammation and oxidant stress. The potential role of PAF-AHs as anticancer enzymes is still controversial since their ability to act both as oncoproteins and tumor suppressor proteins, depending on the metabolized substrate and the targeted cell cycle phase [9]. Anyway, there are several pieces of evidence showing that PAF and related phospholipids can act as tumorigenic agents stimulating proliferation, increasing the expression of anti-apoptotic genes and inducing cell migration [10–12]. Therefore, PAF-AHs, converting platelet-activating factor in a biologically inactive form may decrease PAF levels contrasting the tumorigenesis [9]. There is also evidence suggesting that PAF-AHs can limit multiple key steps involved in the dissemination of tumor cells [13].

In the present study, we characterize the asparaginase and PAF-acetylhydrolase activities of purified human ASPG exploring its effect on the viability of leukemia cells.

## Materials and methods

### Recombinant vectors

The cloning of the full-length cDNA of ASPG in pGEX-4T3 was reported elsewhere [3]. pGEX-4T3-ASPG was used as a template to generate the catalytically inactive point mutant pGEX-4T3-ASPG (T19A) [2] by site-directed mutagenesis (overlap extension PCR) using Pfu (Promega) enzyme and the following oligonucleotides: 5'-GGCGGCACCGCCGGCATGCGG-3' and 5'-CCGCATGCCGGCGGTGCCGCC-3'.

The amplified DNA fragment was then subcloned into pGEX-4T3 at EcoRI and XhoI sites, downstream of the GST tag. All plasmids were checked by sequence analysis.

### Expression and purification of GST, GST-ASPG and GST-ASPG (T19A)

The vectors pGEX-4T3, pGEX-4T3-ASPG and pGEX-4T3-ASPG (T19A) were used to transform E.coli BL21 cells. Bacteria were exponentially grown at 37°C in LB medium supplemented with 100 μg/mL ampicillin and subsequently induced to express fusion proteins by 0.5 mM isopropyl-thiogalactopiranoside (IPTG) for 3h at 37°C. Cells were then centrifuged at 4.000 x g for 15 min, washed with 1X cold phosphate-buffered saline (PBS), resuspended with lysis buffer containing 1% triton X-100, 1% glycerol, 10% sarkosyl, 4 mg/mL lysozyme, 20 mM CHAPS, 10 mM NaF, 2 mM orthovanadate in cold PBS 1X supplemented with protease inhibitors (Roche Molecular Biochemicals) and finally sonicated three times for 30 seconds using a Hielscher UP50H sonicator (0.5 cycles, amplitude 100). Fusion proteins were purified from crude *E*.*coli* extract by single-step affinity chromatography using Glutathione Sepharose® 4B-beads (GE Healthcare) according to the manufacturer's instructions and finally dialyzed at 4°C with 1 mM EDTA, 50 mM NaCl, 50 mM Tris/HCl pH 8 buffer. The concentration of each purified GST-fusion protein was estimated by Coomassie Brilliant Blue R-250 staining using a standard curve of BSA.

### Asparaginase and glutaminase assays

Nesslerization procedure was applied for the determination of ammonia liberated upon deamination of L-asparagine. Different dilutions of recombinant GST-protein were incubated with 25 mM of Tris/HCl pH 8.6 and L-asparagine (concentration depending on the assay) at 37°C for 30 min. The reaction was stopped adding trichloroacetic acid. Ammonia concentrations was evaluated by reading the absorbance at 436 nm after 5 min of Nessler's reagent addition in a microplate reader (Thermo Fisher). The ammonia produced in the reaction was determined by comparing with a standard curve obtained with ammonium sulfate. One international unit (IU) of the enzyme was defined as the amount of the enzyme that liberates one μmole of ammonia per min under the assay conditions. Glutaminase activity was determined following the procedure described above using 15 mM of L-glutamine instead 15 mM of L-asparagine. All experiments were performed in triplicate and repeated more times with different preparations of the recombinant proteins.

### PAF acetylhydrolase assay

The PAF-AH activity of recombinant GST-fusion proteins was detected using PAF acetylhydrolase Assay Kit (Abcam) that can detect free thiol groups that are released upon hydrolysis of the acetyl thioester bond at the sn-2 position of 2-thio PAF, the modified substrate used in the assay. The reaction was started adding a range of 10–60 nM of purified GST-fusion proteins [GST, GST-ASPG and GST-ASPG (T19A)] to the sample mixture containing 200 μM 2-thio-PAF, 0.1 mM of DTNB and 100 mM Tris/HCl pH 7.2. The product of the reaction catalyzed by PAH-AH reacts with DNTB forming 5-thio-2-nitrobenzoic acid, a compound that has a maximum absorbance peak at 412 nm. The absorbance, evaluated by a microplate reader (Thermo Fischer) of 5-thio-2-nitrobenzoic acid was used to calculate the International Unit of PAF-AH activity of our recombinant proteins. All experiments were performed in triplicate and repeated more times with different preparations of the recombinant proteins.

### Cell culture

K562 (Human immortalized myelogenous leukemia), NALM-6 (Human B cell precursor leukemia) and MOLT-4 (human T lymphoblast; acute lymphoblastic leukemia) cell lines were cultured in RPMI-1640. PBMCs were isolated from blood by density gradient centrifugation using Biocol Separating Solution (Biochrom GmbH), and cultured in RPMI supplemented with 4% of phytohemagglutinin (Life Technology). Human Dermal Fibroblasts-adult (HDFa) cells were cultured in DMEM. All the media were supplemented with 10% of Fetal Bovine Serum (FBS) and 1% penicillin/streptomycin (from stock solution of 10.000 units penicillin and 10 mg streptomycin per mL). The cells were grown at 37°C in a 5% CO_2_ atmosphere incubator.

### Cytotoxicity assay

Leukemia cell lines and PBMCs were seeded in 96-well plates at a density of 1x10^4^ cells/well, HDFa cells were seeded at a density of 5x10^3^cells/well. All the cells were incubated with different concentrations of purified recombinant proteins for 24h. Microscopy was performed on a Leica DMI400B automated Inverted Microscope, images were captured using a Leica DFC 350 FX camera (10X Magnification) and acquired by Leica Application Suite Software (Version 2.8.1).

Cells were then treated with the CCK8 reagent (Sigma), following manufacturer's instructions. Cell viability was evaluated by determining the concentration of WST-8-formazan, a compound with a typical absorbance at 450 nm, which formation is directly proportional to the number of living cells. Absorbance was read by using a microplate reader (Thermo Fisher). The viability of cells was also evaluated by the Trypan Blue exclusion assay. Experiment of comparison of cytotoxic activity between GST-ASPG and bacterial Asparaginase was performed by using *E*. *Coli* asparaginase (Sigma). Cytotoxicity of GST-ASPG on leukemia cells was also evaluated in the presence of D-asparagine (200 mM), L-asparagine (50 mM) and PAF (10μM), using the CCK8 assay.

### Caspase assay

To allow direct determination of the percent of live, apoptotic, dead and necrotic populations, distinguished by the presence or absence of activated caspases and/or an intact plasma membrane [14], 2x10^4^ K562 cells/mL, treated with 700 ng of GST, GST-ASPG and GST-ASPG (T19A) for 6, 12 and 24h, were used for Guava Caspase Assay according with the manufacturer’s instructions (Millipore, 4500–0500). Four populations of cells were thus distinguished in this assay: Lower-left quadrant: viable cells (negative for both Caspase and 7-AADreagents); Lower-right quadrant: cells in the middle stages of apoptosis (positive for Caspase Reagent and negative for 7-AAD); Upper-right quadrant: cells in the late stages of apoptotic or dead (Positive for both Caspase and 7-AAD reagents); Upper-left quadrant: necrotic cells (negative for Caspase Reagent and positive for 7-AAD reagent).

### Statistical analysis

GraphPad Software Prism 5 was used for regression and graphical analysis. To determine the statistical significance of each effect student’s T-test or the one-way analysis of variance (ANOVA) test followed by Bonferroni post-test for multiple comparisons was used. Statistical significance was set at *p* < 0.05.

## Results

### L-asparaginase activity of ASPG

**Fig 1:**
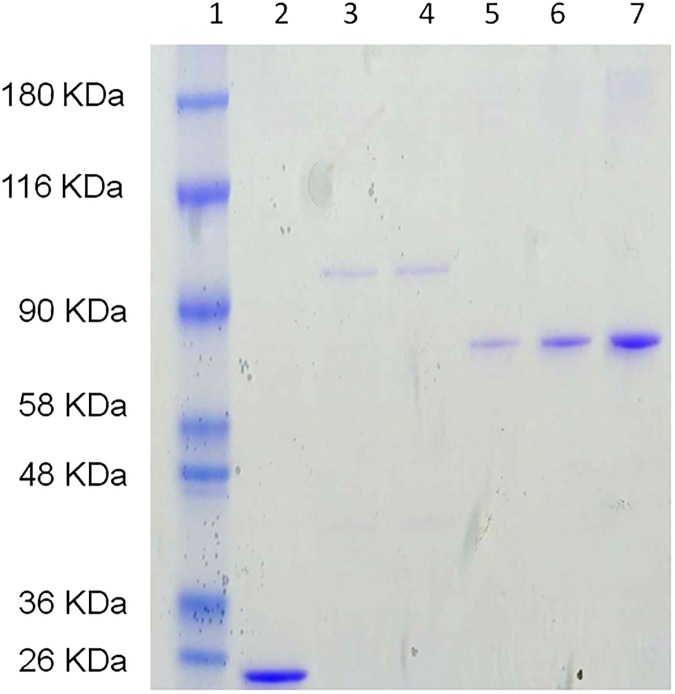
SDS Page of purified GST-fusion proteins. Line 1: marker, line 2: GST, line 3: GST-ASPG, line 4: GST-ASPG (T19A), line 5–7: 50, 100 and 250 ng of bovine serum albumin respectively.

Recombinant ASPG and ASPG (T19A) mutant fused to GST were induced and purified as described in Materials and Methods. The proteins were checked by SDS-PAGE (Fig 1). The catalytic domain of ASPG is located in its N-terminal region and the threonine at position 19 is a residue directly involved in all catalytic activities of ASPG (http://www.uniprot.org/uniprot/Q86U10), including that of L-asparaginase [2].

**Fig 2:**
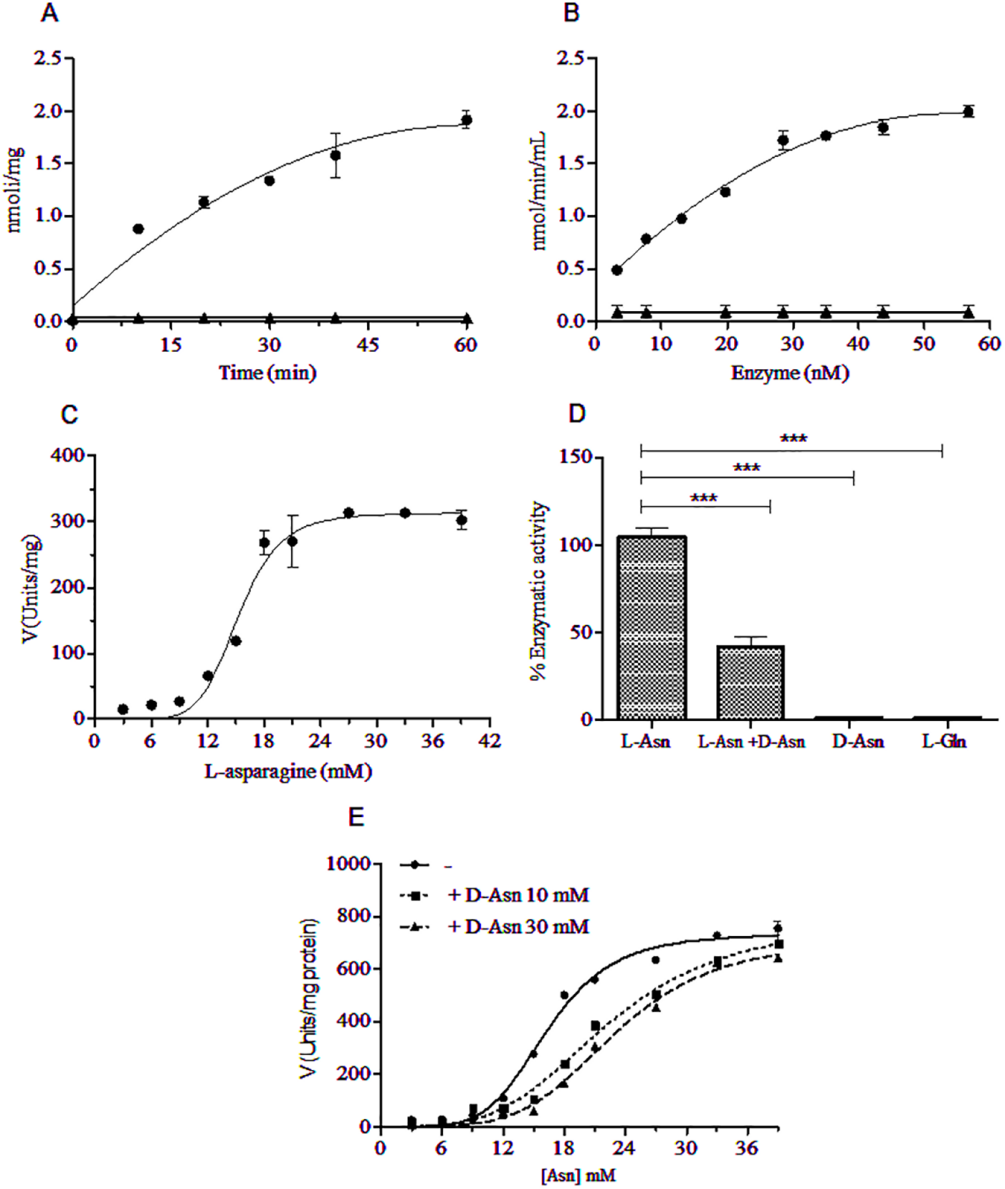
Characterization of L-asparaginase activity of GST-ASPG. L-Asparaginase activity of GST-ASPG (●) and its catalytically inactive mutant GST-ASPG (T19A) (▲) was evaluated by Nessler’s method in a time dependent manner (A), as a function of enzyme concentration (B) and substrate concentration (C). A hill slope of 6.9 and an S_0.5_ value of ≈13 mM were estimated. Ammonia release of GST-ASPG was measured using 15 mM of L-asparagine alone or in presence of 15 mM of D-asparagine; No detectable enzymatic activity was found using 15 mM of D-asparagine or 15 mM of L-glutamine as substrates (D). Steady state kinetic of recombinant GST-ASPG was calculated in presence of 0 mM (●), 10 mM (■) and 30 mM (▲) of D-asparagine and a Ki value of 71 mM was estimated (E). All the experiments were performed at 37°C as reported in Materials and Methods using 1.5 μg of each protein; data points are represented as means ± SD of triplicate sample measurements. *** (*p* < 0.001).

Asparaginase activity of GST-ASPG and GST-ASPG (T19A), tested by Nessler method in function of time (Fig 2A) and enzyme concentration (Fig 2B) revealed a linear range of ammonia released with R-squared values of 0.95 and 0.98 respectively. Data points were fitted with a second order polynomial equation. No asparaginase activity was detected for GST-ASPG (T19A).

The steady-state kinetic measured in a range of 3–39 mM of L-asparagine for 30 min at 37°C, revealed that the enzyme did not follow the Michaelis-Menten kinetic, instead it exhibited a sigmoidal kinetic behavior, a hallmark of an allosteric enzyme. The kinetic data were fitted using the Hill equation. A Hill slope of 6.9 and an S_0.5_ value of ≈13 mM were estimated (Fig 2C).

The asparaginase activity of GST-ASPG was measured in the presence of different compounds. A general decrease of activity was detected except for MgCl_2_ that was able to confer a moderate increase of the asparaginase activity as shown in Table 1.

**Table 1:** Effect of different compounds on the activity of GST-ASPG.

Compound	% residual activity
-	100
MgCl_2_	137.21 ± 2,82
EDTA	71.98 ± 2,35
DTT	44.92 ± 0,75
ZnCl_2_	60.56 ± 1,62
KCl	61.04 ±1.32
CaCl_2_	58.89 ± 5.02
FeSO_4_ · 7 H_2_O	47.42 ± 1,73
BSA	17.68 ± 0.55

1.5 μg of purified GST-ASPG were pre-incubated with different divalent metal ions, EDTA and DTT (at a final concentration of 1mM) for 10min at 37°C. Subsequently, samples were tested for residual L-asparaginase activity by Nessler’s method as described in Materials and Methods. Data are shown as means ± SD of triplicate measurements.

L-glutamine and D-asparagine, the enantiomer of the physiological substrate of GST-ASPG, were not metabolized (Fig 2D) and the kinetic study of the inhibition showed that D-asparagine behaves as a competitive inhibitor with a Ki value of 71 mM (Fig 2E).

### PAF acetylhydrolase activity of ASPG

**Fig 3:**
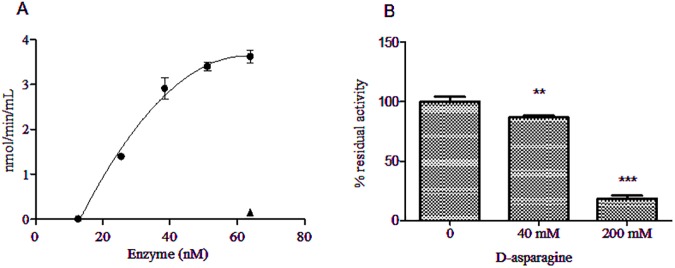
PAF-AH activity of GST-ASPG. (A) The PAF-AH activity of recombinant GST-ASPG (●) and its point mutant GST-ASPG (T19A) (▲) was measured as function of enzyme concentration using Abcam's PAF acetylhydrolase Assay Kit as reported in Materials and Methods. Data points are represented as means ± SD of triplicate sample measurements and were fitted with a second order polynomial equation in GraphPad Prism software. (B) The residual PAF-acetylhydrolase activity of GST-ASPG was measured after pre-incubation for 10 min of 1.5 μg of GST-ASPG with 40 mM and 200 mM of D-asparagine using Abcam's PAF-AH assay. Data are shown as means ± SD of triplicate measurements. ** (*p* < 0.01); *** (*p* < 0.001).

PAF acetylhydrolase activity was determined by means of free thiol groups, released from hydrolysis of the PAF acetyl thioester as detailed in Materials and Methods. A dose-dependent PAF acetylhydrolase activity, with an R-squared value of 0.99, was detected in GST-ASPG but not in the GST-ASPG (T19A) mutant (Fig 3A). Data points were fitted with a second order polynomial equation. Interestingly, the PAF-AH activity of GST-ASPG was also significantly inhibited by D-asparagine at concentrations higher than those used to block L-asparaginase activity (Fig 3B).

### GST-ASPG is cytotoxic for leukemic cells

**Fig 4:**
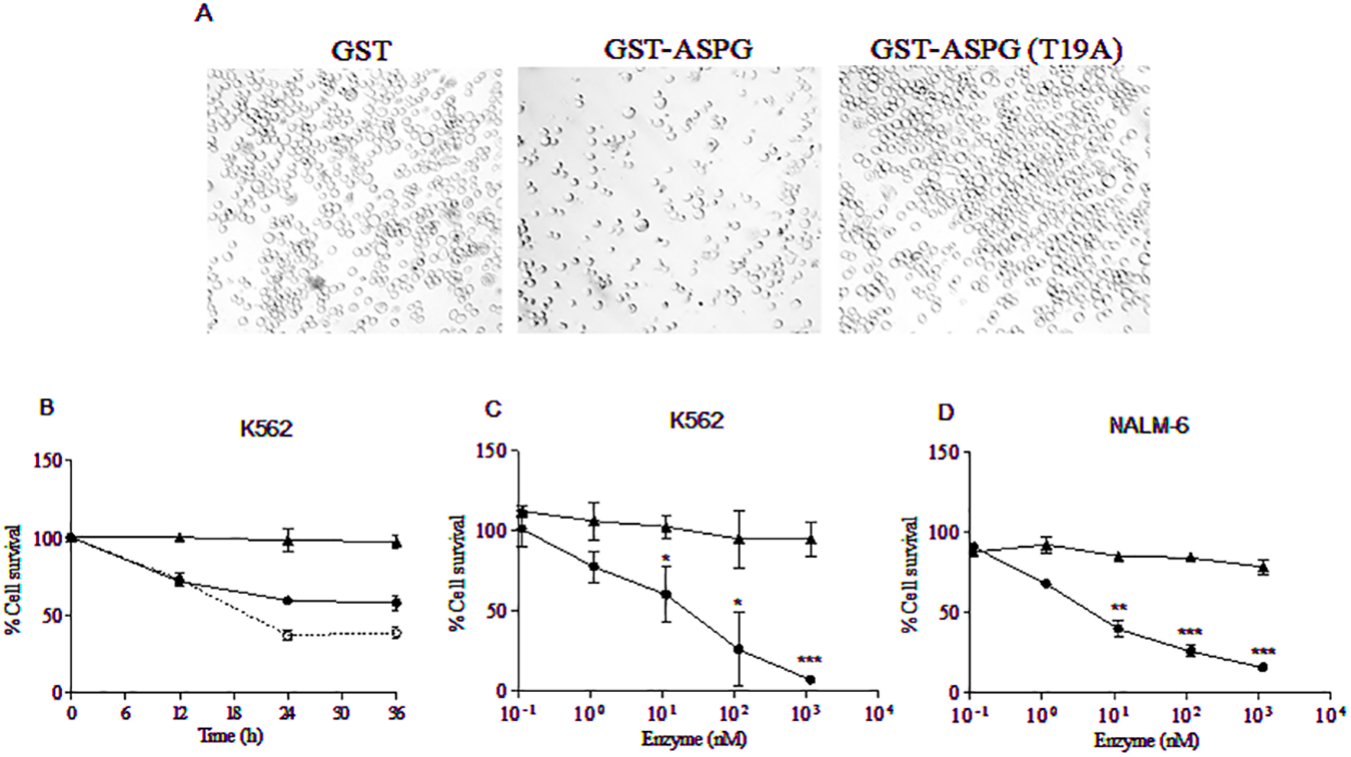
Cell growth inhibition by GST-ASPG. (A) K562 cells after 24h of incubation with 1000 ng (115 nM) of GST-ASPG and its inactive mutant GST-ASPG (T19A). (B) Time course of K562 cells treatment with 115 nM of GST-ASPG (T19A) (▲), 115 nM of GST ASPG (●) and after further addition of 115 nM of GST-ASPG at 12h (ο).Viability of K562 (C) and NALM-6 (D) cells after 24h of treatment with increasing concentrations of GST-ASPG (●) and its inactive catalytically mutant GST-ASPG (T19A) (▲). The results (the average and the standard deviation of three independent experiments) were evaluated by Trypan Blue exclusion assay and fitted using GraphPad Prism software. * (*p* < 0.05); ** (*p* < 0.01); *** (*p* < 0.001).

Asparaginase and PAF acetylhydrolase activities are both involved in cell growth inhibition [5, 9]. In particular, the asparaginase activity is mainly effective on the growth inhibition of leukemic cells. Thus, as usually performed with bacterially derived asparaginases, leukemic cells were treated with purified GST-ASPG added to the cellular medium. Cytotoxicity of GST-ASPG in K562 cells was evaluated by cell counting using the trypan blue exclusion method in a time-course experiment. Cell counts of K562 cells treated with GST-ASPG decreased significantly after 24h in comparison with GST-treated control cells (Fig 4A and 4B). No significant decrease in cell counts was detected in K562 treated with GST-ASPG (T19A) mutant protein, indicating that the catalytic activity of ASPG is required for its cytotoxic action. Since the time course of cytotoxicity suggested an initial higher cytotoxic activity of GST-ASPG on K562, a sequential dose of the enzyme was added to the medium in order to investigate whether GST-ASPG decreased its activity during the time or was induced a selection of K562-resistant cells. The addition of more GST-ASPG after 12h further increased its cytotoxic effect on K562 cells indicating that fresh enzyme was able to kill additional cells (Fig 4B). This behavior appears to be consistent with a possible decrease of GST-ASPG activity during the time whereas the selection of resistant cells appears less probable.

Next, the cytotoxicity of GST-ASPG protein was evaluated by treating K562 and NALM-6 cells with increasing concentrations of GST-ASPG and cell counting after 24h. GST-treated cells were used as a control. A dose-dependent decrease in cell counts was detected in cells treated with GST-ASPG. No significant decrease was instead observed in leukemic cells treated with the GST-ASPG (T19A) mutant protein (Fig 4C and 4D).

**Fig 5:**
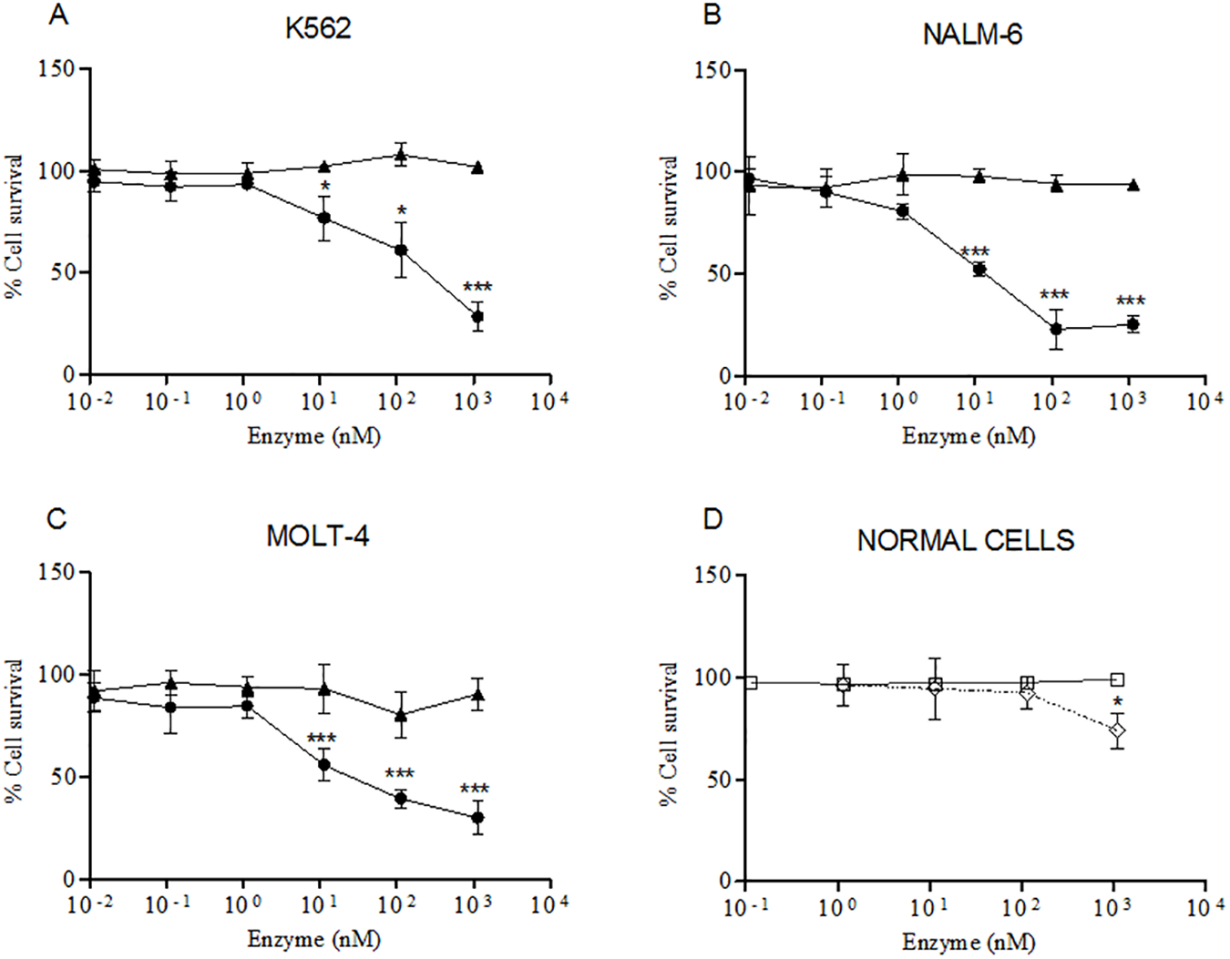
Cytotoxicity of GST-ASPG on human leukemia cell lines and normal cells. The percentage of cell survival was evaluated by CCK8 assay in K562 (A), NALM-6 (B) and MOLT-4 (C) cell lines after 24h of treatment with GST-ASPG (●) and its inactive catalytically mutant GST-ASPG (T19A) (▲). HDFA (□) and PBMCs (◊) were used to analyze the cytotoxicity of GST-ASPG in normal cells (D). The results were fitted using GraphPad Prism software and represent the average and the standard deviation of three independent experiments. * (*p* < 0.05); *** (*p* < 0.001).

To further assess the cytotoxic activity of GST-ASPG cell viability was evaluated by using the CCK8 reagent. Leukemia K562, NALM-6 and MOLT-4 cell lines were treated with increasing concentrations of GST-ASPG and the viability of treated-cells was then measured and compared with GST- treated cells (Fig 5A–5C).The use of CCK8 reagent confirmed the cytotoxicity of GST-ASPG in a dose dependent manner. The cytotoxicity curves obtained were fitted with the IC50 equation. IC50 values for K562, NALM-6 and MOLT-4 were 120 nM, 15.2 nM and 20.9 nM, respectively (S1 Fig).

Again, in cells treated with GST-ASPG (T19A), no significant reduction in cell viability was detected, similarly to what obtained in cell counting experiments.

As a control, PBMCs and HDFa (adult human dermal fibroblasts) cells were treated with GST-ASPG. In both cases, no reduction of cell viability was detected in comparison with GST-treated cells with the exception of PBMCs treated with the highest concentration of GST-ASPG used (10^3^ nM), in which a slight cytotoxicity was detected (Fig 5D). However, in PBMCs, the lowest cytotoxic concentration of GST-ASPG was two order of magnitude higher than that in leukemia cells. We also performed in K562 cells a comparison of cytotoxic potential between ASPG and *E*. *Coli* Asparaginase and similar cytotoxic activity was found (S2 Fig).

**Fig 6:**
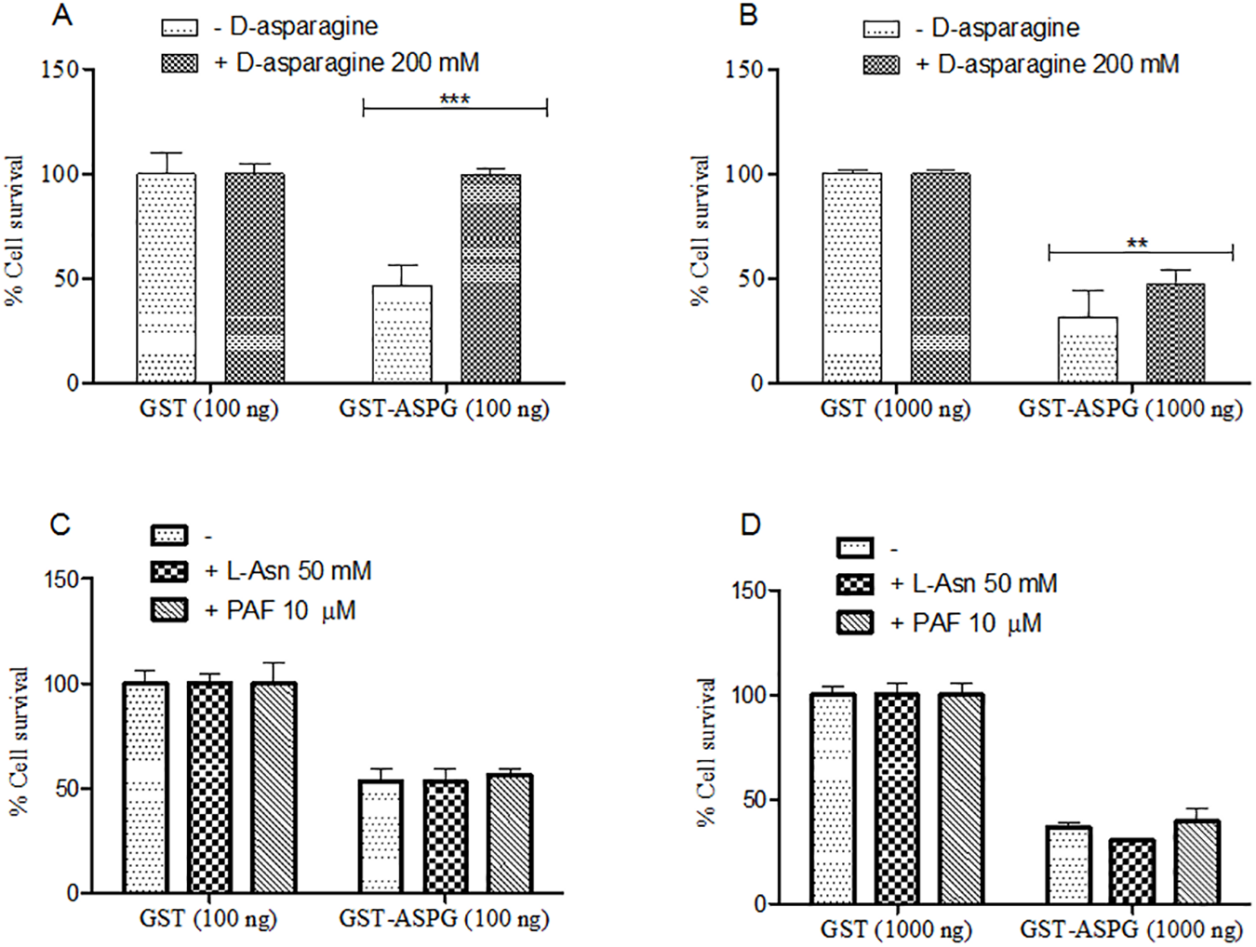
Cytotoxicity of GST-ASPG on K562 cell line in presence of D-asparagine, L-asparagine and PAF. Data show the effects of 24h of treatment with 100 ng (A) or 1000 ng (B) of GST-ASPG in the presence of 200 mM of D-asparagine, 50 mM of L-asparagine and 10 μM of PAF (C and D) in the culture medium. The percentage of cell survival was evaluated by CCK8 assay and data represent means ± SD of triplicate measurements. ** (*p* < 0.01) *** (*p* < 0.001).

In order to block the cytotoxic effect of GST-ASPG in K562 cells D-asparagine was added to the culture medium. We observed that, after 24h, a higher concentration of D-asparagine (200 mM) completely abolished the cytotoxic activity of 100 ng (11.5 nM) of GST-ASPG (Fig 6A) and significantly decreased the cytotoxicity of 1000 ng (115 nM) of GST-ASPG (Fig 6B). To investigate the cytotoxic potential of ASPG, supranormal levels of ASPG substrates were added to the culture medium of K562 cells. Neither the addition of L-asparagine or PAF influenced significantly the ASPG cytotoxic activity (Fig 6C and 6D). In untreated K562 cells, in our experimental conditions, limited cytotoxicity was observed with the addition in the medium of D-asparagine 200 mM, L-asparagine 50 mM or PAF 10 μM (data not shown).

**Fig 7:**
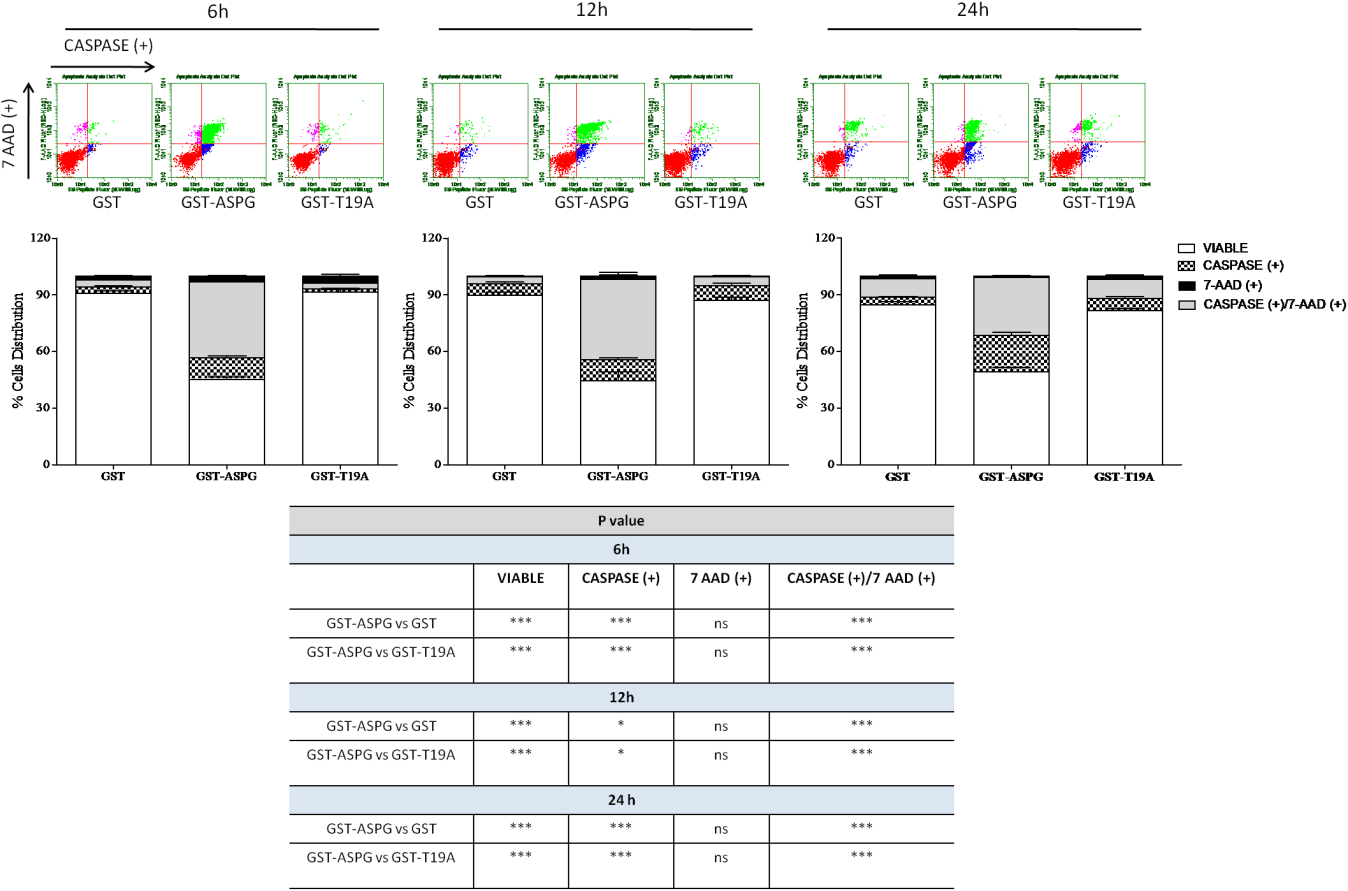
Caspase assay on K562. (A) Representative Guava caspase assay graphs of K562 cells lines treated with 0.7 μg of GST, GST-ASPG or GST-ASPG (T19A) for 6, 12 and 24h. (B). Bar graphs representing the percentage of caspase and/or 7AAD positive cells after treatment with 0.7 μg of GST, GST-ASPG or GST-ASPG (T19A) for 6, 12 and 24h. (C) Table representing the statistical significances between groups. *(p < 0.05); **(*p*< 0.01); ***(*p*< 0.001).

Finally, to characterize the possible mechanism of ASPG-dependent cell death [15], a caspase assay was performed. This particular assay allows the distinction between early stage apoptosis [Caspase Reagent(+) and 7-aminoactinomycin D (7-AAD)(-)] and late stage apoptosis or death [Caspase Reagent(+) and 7-AAD(+)]. A clear apoptotic death was detected in cells treated with GST-ASPG in comparison with controls [GST and GST-ASPG(T19A)]. Interestingly, a late phase apoptotic death was found in the earlier time points, whereas at 24h a relative increase of early apoptotic death was detected, suggesting a progressive decrease in the efficiency of GST-ASPG (Fig 7A–7C).

## Discussion

The present study focuses on the cytotoxic effect on Leukemia cell lines of ASPG, a human protein previously characterized as an asparaginase [2] and lysophospholipase [3] enzyme. Based on the sequence analysis, ASPG displays also acetylhydrolase activity on the platelet-activating factor (PAF) similar to that detected in the rat homolog 60kDa Lysophospholipase [1]. The catalytic domain responsible for multiple enzymatic activities is located in the N-terminal region of the protein.

The biochemical characterization of the asparaginase activity of human ASPG revealed that it exhibits a relatively low affinity for L-asparagine (Km in the millimolar range), while bacterial enzymes, that are commonly used as anticancer drugs, have a higher affinity for the substrate. Plotting the kinetic of the asparaginase activity of ASPG versus substrate concentration we obtained a sigmoidal curve that suggests an allosteric regulation of the enzyme.

Our biochemical findings are in agreement with those obtained by Karamitros and Konrad [2] who characterized the N-terminal region of ASPG (residues 1 to 369) showing that this part of the protein forms a stable and functional folding unit in the absence of the C-terminal ankyrin repeat domain.

Similarly to what observed in some bacterial asparaginases [16], a significant increase in the asparaginase activity of ASPG in the presence of MgCl_2_ was detected, even if this salt did not modify the Km value (data not shown). On the other hand, significant inhibition of asparaginase activity was observed in the presence of D-asparagine.

Human ASPG shares a percentage of identity greater than 80% and a percentage of similarity of 95% with gpASNase1, the guinea pig asparaginase which was identified as the main responsible for the capacity of the guinea pig serum to kill lymphoma cells. Despite this similarity, there are different kinetic properties between human ASPG and gpASNase. In fact, ASPG has a higher Km value for asparagine, probably attributable to the presence of two amino acids in proximity to the catalytic threonine 19 and to the allosteric regulation [17].

PAF-AH activity was detectable in ASPG and the catalytic activity depends on the threonine at position 19. The PAF-AH activity was also inhibited by D-asparagine at a concentration higher than that required to block L-asparaginase activity.

Testing the effect of ASPG on leukemia cell lines, a significant cytotoxicity of recombinant ASPG was found. In leukemia cells treated with the T19A catalytic inactive mutant, no significant reduction of cell viability was detected, demonstrating that the enzymatic activity is essential for the cytotoxicity of ASPG. Experiments performed in PBMCs and HDFa (adult human dermal fibroblasts) show negligible effects of ASPG on the viability, demonstrating that the cytotoxic activity of ASPG is selective for leukemia cell lines.

We and others [2] did not detect in ASPG any glutaminase activity, that seems to be essential for the cytotoxicity of bacterial asparaginases [18–19]. However, a mutant of *Erwinia chrysanthemi* L-asparaginase with very low glutaminase but relevant cytotoxic activity has been recently engineered [20]. As in bacterial asparaginases, the cytotoxicity of human ASPG could be dependent on other enzymatic activities, besides asparaginase. We argue that, in our case, the PAF-AH activity could also play a relevant role in the cytotoxic potential of ASPG.

PAF-AHs catalyze the removal of the acetyl group at the sn-2 position of PAF to generate the inactive lyso-PAF and acetate, thus regulating the levels of lipid mediators that appear to play a role in initial tumorigenic events and/or spreading of the disease [21]. Anti-neoplastic activities of unnatural ether-linked phospholipid analog, structurally related to lyso-PAF, was characterized for leukemia cells [22]. Dupuis et al. demonstrated that K562 cell line can produce, metabolize and use PAF to stimulate the thymidine incorporation thus favoring cell growth and erythropoiesis. They also demonstrated that those effects are mediated by PAF receptor [23].

In our experiments, lymphoid cells (NALM-6 and MOLT-4) resulted to be more sensitive than myeloid cells (K562) to ASPG treatment. We hypothesize that the relative resistance of K562 to ASPG cytotoxicity could be mechanistically attributed to the asparaginase and PAF-acetylhydrolase activities of ASPG. Indeed, there is evidence demonstrating that K562 cells are more resistant to asparagine deprivation [19] and release more PAF compared with lymphoid cells [24].

Interestingly, D-asparagine blocked ASPG cytotoxic activity in K562 cells at a concentration that inhibits either asparaginase or PAF-AH activities, indicating that both enzymatic activities of ASPG contribute to its cytotoxicity.

The addition of substrate supranormal levels of either L-asparagine or PAF did not significantly modify ASPG cytotoxic effects. Finally, our results demonstrated that GST-ASPG induced apoptosis in K562 cells by a caspase-dependent pathway. Apoptosis is generally characterized by distinct morphological and biochemical events which serve to distinguish it in two main phases: the early phase, in which there is the activation of effector caspases, and late phase, in which there are the cleavage of PARP and the internucleosomal DNA fragmentation and the exposure of phosphatidylserine on the external surface of plasma membrane [25].

More specifically, the time course of cells treated with GST-ASPG revealed that ASPG could act through a biphasic mechanism, in fact, a late phase of apoptosis was observed in earlier time of treatment (positivity to both caspase and 7-AAD reagents) whereas an early phase was observed later (positivity only for the caspase reagent). Probably, this mechanism could be due to a gradual loss of enzyme activity of ASPG once it is added to the medium of the cell culture. Therefore, ASPG, losing its cytotoxic potential, would not be able to induce apoptosis in K562 with the same initial power. Our interpretation is further confirmed by the experiment in which we observed an increase of cytotoxicity upon a further addition of ASPG after the initial treatment.

**Fig 8:**
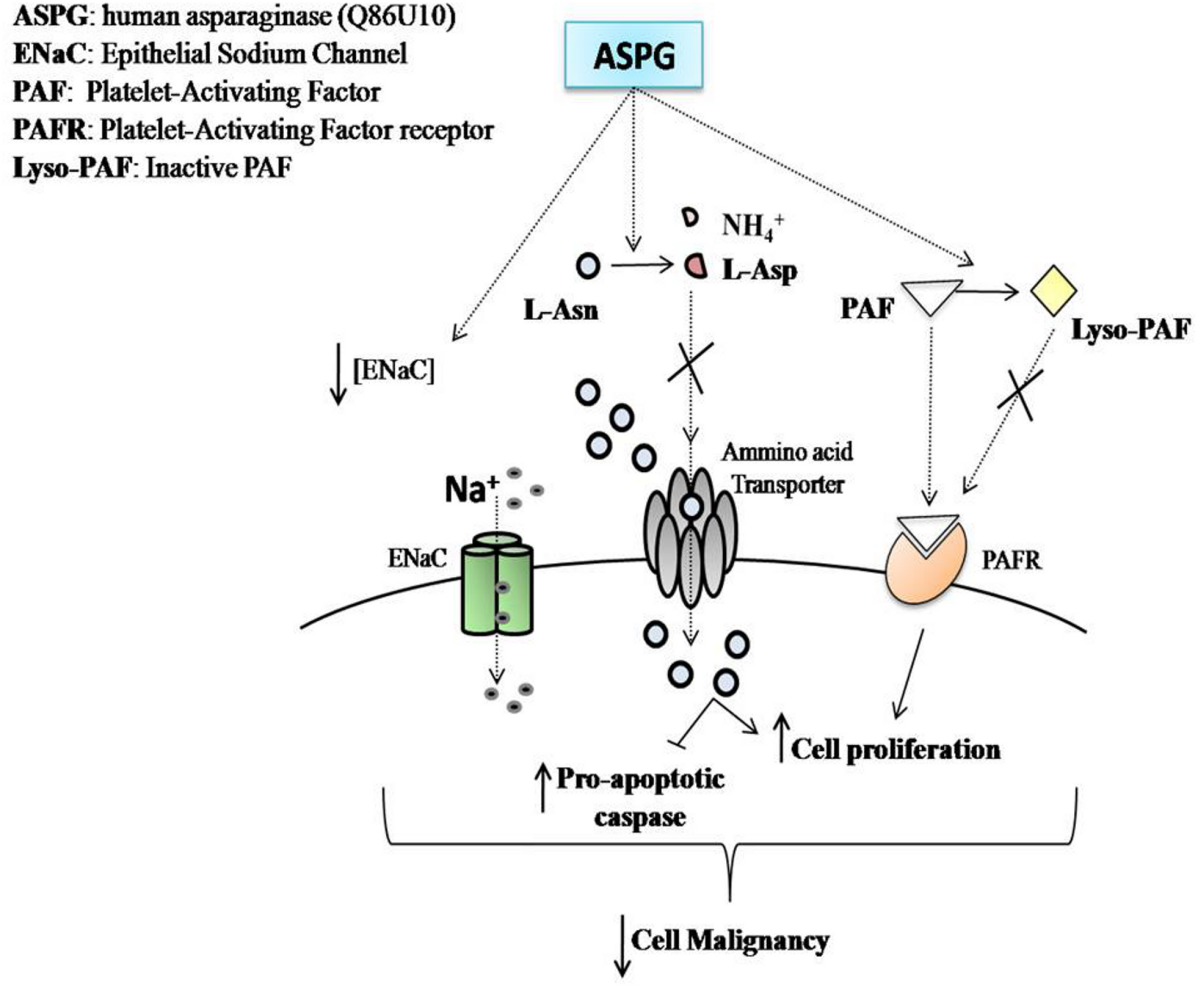
Schematic representation of ASPG action on the growth and survival of leukemia cells. ASPG with its L-asparaginase activity could deprive leukemia cells of L-asparagine, which is an essential metabolite for its malignant growth, inducing apoptosis and blocking cell proliferation. ASPG could also inducing the arrest of cell proliferation converting the active PAF in the inactive form Lyso-PAF and down-regulating the expression of the Epithelial Sodium Channel [3, 26–27].

In conclusion, we demonstrate that ASPG has a cytotoxic activity against leukemic cells. ASPG shows a relatively high Km for L-asparagine, nevertheless, it shows a PAF-AH activity that can be able to cooperate with the asparaginase activity to inhibit the growth and inducing the apoptotic response of leukemic cells. These features reveal that ASPG may be an interesting candidate for new therapeutic approaches against leukemia (Fig 8).

## Supporting information

S1 Fig Ratios of the IC50 of GST-ASPG on growth of leukemia cells. IC50 values were calculated using GraphPad Software after 24h of incubation of K562 (A), NALM-6 (B) and MOLT-4 (C) cell lines with increased concentrations of GST-ASPG.
(TIF)

S2 Fig Comparison between GST-ASPG and E. coli asparaginase on K562 cell viability. The percentage of cell survival was evaluated by CCK8 assay using increasing concentration of GST-ASPG (●) and E. coli Asparaginase (ο). Results were fitted using GraphPad Prism software and represent the average and the standard deviation of three independent experiments.
(TIF)
