# Supplementary figures and images for: The human asparaginase enzyme (ASPG) inhibits growth in leukemic cells
Source: PLoS One. 2017 May 24;12(5):e0178174. doi: 10.1371/journal.pone.0178174 (PMC5443537; doi:10.1371/journal.pone.0178174)

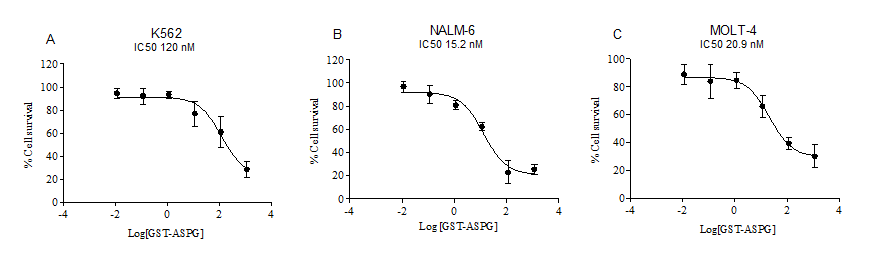

Supplement: S1 Fig — IC50 values were calculated using GraphPad Software after 24h of incubation of K562 (A), NALM-6 (B) and MOLT-4 (C) cell lines with increased concentrations of GST-ASPG. (TIF) [file pone.0178174.s001.tif]

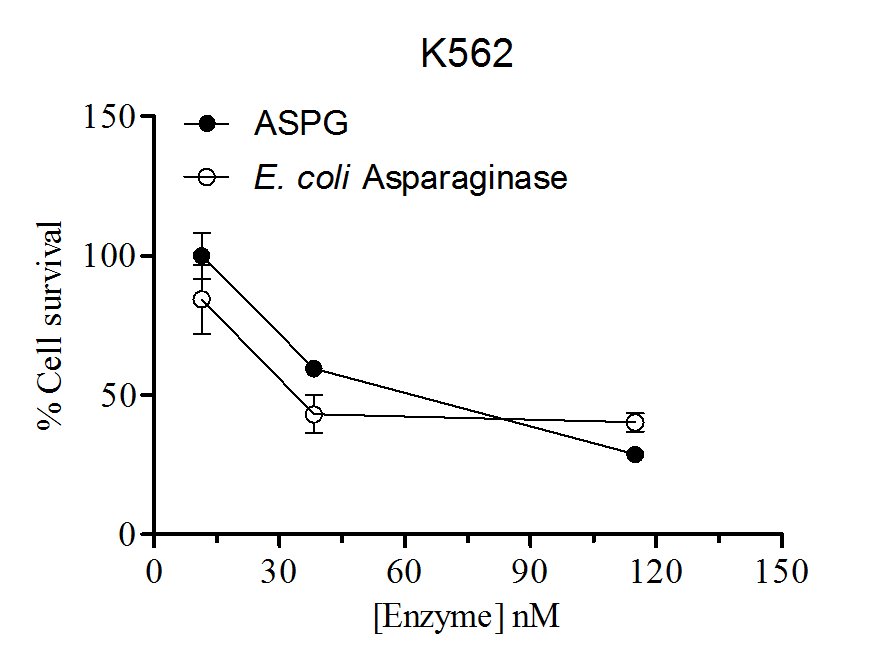

Supplement: S2 Fig — The percentage of cell survival was evaluated by CCK8 assay using increasing concentration of GST-ASPG (●) and E. coli Asparaginase (ο). Results were fitted using GraphPad Prism software and represent the average and the standard deviation of three independent experiments. (TIF) [file pone.0178174.s002.tif]
